# Transcranial Pulse Stimulation in Parkinson's Disease: A Systematic Review

**DOI:** 10.1002/brb3.71895

**Published:** 2026-11-03

**Authors:** Gabrielly Santos Pereira, Gustavo Henrique de Mello Rosa, Palmerindo Antônio Tavares de Mendonça Neto, João Eduardo de Araújo, Marcelo Lourenço da Silva

**Affiliations:** ^1^ Department of Health Sciences Neuropsychobiology and Motor Control Laboratory, Ribeirão Preto Medical School University of São Paulo Ribeirão Preto São Paulo Brazil; ^2^ Instituto Regenera Dor Juazeiro do Norte Ceará Brazil; ^3^ Laboratory of Neuroscience, Neuromodulation and Study of Pain (LANNED) Federal University of Alfenas (UNIFAL‐MG) Alfenas Minas Gerais Brazil

**Keywords:** motor function, neuroplasticity, noninvasive brain stimulation, Parkinson's disease, transcranial pulse stimulation, ultrasound neuromodulation

## Abstract

**Background:**

Transcranial pulse stimulation (TPS) is an emerging ultrasound‐based neuromodulation technique with potential applications in Parkinson's disease (PD). This systematic review evaluated its clinical efficacy, neurophysiological and neuroimaging effects, safety, and certainty of evidence.

**Methods:**

PubMed/MEDLINE, Embase, and Scopus were systematically searched for clinical studies investigating TPS in adults with idiopathic PD. Motor, non‐motor, cognitive, neurophysiological, neuroimaging, and safety outcomes were narratively synthesized because of methodological heterogeneity. Risk of bias was assessed, and evidence certainty was evaluated using the Grading of Recommendations Assessment, Development, and Evaluation (GRADE) framework.

**Results:**

Four studies involving 80 participants were included: two sham‐controlled studies, one prospective open‐label pilot study, and one retrospective uncontrolled study. Uncontrolled studies reported improvements in motor function, non‐motor symptoms, cognition, mood, and quality of life. However, a randomized double‐blind crossover trial demonstrated no superiority of active TPS over sham for global motor scores (MDS‐UPDRS Part III). Significant improvements in left‐hand manual dexterity persisted for one month and were accompanied by sensorimotor activation and white‐matter changes. A single‐session study reported immediate resting‐tremor reduction, although sustained superiority over sham remained uncertain. Electroencephalographic findings suggested cortical oscillatory modulation. No serious treatment‐related adverse events were reported. Evidence certainty ranged from moderate to very low.

**Conclusions:**

TPS shows preliminary potential to modulate sensorimotor networks and improve selected motor outcomes in PD. However, evidence remains insufficient to establish overall clinical efficacy. Larger, rigorously controlled trials are required to determine sustained benefits, optimal stimulation protocols, and long‐term safety.

## Graphical abstract

Transcranial pulse stimulation (TPS) may modulate sensorimotor networks in Parkinson's disease and improve selected outcomes, particularly manual dexterity and short‐term resting tremor. However, the certainty of evidence ranges from moderate to very low, and current evidence is insufficient to establish routine clinical efficacy.

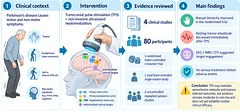

## 1 Introduction

Parkinson's disease (PD) is a progressive neurodegenerative disorder and one of the most rapidly growing causes of neurological disability worldwide (Ben‐Shlomo et al. 2024). Its clinical expression extends beyond bradykinesia, rigidity, tremor, and progressive gait and postural impairment to include cognitive, neuropsychiatric, autonomic, sleep, fatigue, and pain‐related manifestations (Bloem et al. 2021; Takamatsu et al. 2018). The cumulative burden of these non‐motor symptoms is strongly associated with functional disability and reduced health‐related quality of life (Martinez‐Martin et al. 2011; Müller et al. 2013).

Current treatments remain predominantly symptomatic. Dopaminergic therapies provide substantial motor benefit but are limited over time by motor fluctuations, dyskinesias, and progressive symptoms that become less responsive to dopamine replacement (Foltynie et al. 2024). Deep brain stimulation can improve levodopa‐responsive motor complications in appropriately selected patients but requires surgical implantation and does not prevent progression of axial, cognitive, and other non‐dopaminergic manifestations (Deuschl et al. 2006; Weaver et al. 2009; Fasano et al. 2015). These limitations have stimulated interest in noninvasive approaches capable of modulating dysfunctional motor networks and promoting adaptive neuroplasticity (Madrid and Benninger 2021).

Transcranial ultrasound neuromodulation has emerged as a potential alternative because acoustic energy can be directed to predefined neural targets with high spatial specificity. Transcranial pulse stimulation (TPS) is a distinct ultrasound‐based technique that delivers neuronavigated, ultrashort, single‐cycle, broadband acoustic pulses. TPS‐specific experimental and computational studies support spatially localized intracranial energy delivery, whereas a sham‐controlled study in healthy participants demonstrated measurable changes in brain‐network organization and white‐matter microstructure following TPS (Beisteiner et al. 2020; Truong et al. 2022; Matt et al. 2022). These pulse characteristics distinguish TPS from most transcranial focused ultrasound stimulation approaches, which generally use multi‐cycle sinusoidal bursts at a defined carrier frequency (Truong et al. 2022).

The cellular mechanisms underlying TPS remain incompletely established. Mechanotransduction, modulation of mechanosensitive ion channels, changes in membrane excitability, and downstream neuroplastic responses remain biologically plausible mechanisms (Zhu et al. 2023). Recent TPS‐specific preclinical studies have begun to refine this mechanistic framework. In healthy and Alzheimer's disease mouse models, Karakatsani, Nozdriukhin, et al. (2025) demonstrated reproducible TPS‐induced increases in cerebral microvascular blood volume, whereas the blood–brain barrier remained intact, suggesting modulation of cerebrovascular dynamics without blood–brain barrier opening. In a subsequent study combining fluorescence calcium imaging, resting‐state functional magnetic resonance imaging (fMRI), and c‐Fos immunohistochemistry, TPS induced rapid calcium influx, increased c‐Fos expression in the dentate gyrus, and transient reorganization of functional connectivity across hippocampal, hypothalamic, and other limbic networks; higher stimulation intensities also triggered spreading depolarization waves (Karakatsani, Gezginer, et al. 2025). Together, these findings provide TPS‐specific experimental evidence that ultrashort acoustic pulses can influence both cerebrovascular dynamics and neuronal/network activity. However, these data are preclinical and derive from healthy and Alzheimer's disease mouse models rather than PD, so their relevance to mechanisms operating in patients with PD remains to be established.

Clinical TPS studies have evaluated motor symptoms, tremor, manual dexterity, non‐motor outcomes, cognition, electroencephalographic activity, functional activation, white‐matter microstructure, and safety in PD (Osou et al. 2024; Manganotti et al. 2025; Gianlorenco et al. 2026; Matt et al. 2026). However, the evidence remains heterogeneous in study design, stimulation target, acoustic dose, number of sessions, comparator, and follow‐up. Previous reviews have focused primarily on TPS in Alzheimer's disease or have combined heterogeneous neurological and psychiatric populations (Chen et al. 2024; Polte et al. 2025). To our knowledge, no systematic review has specifically examined TPS in PD while integrating clinical efficacy, stimulation parameters, neurophysiological and neuroimaging target engagement, safety, risk of bias (RoB), and certainty of evidence. Therefore, this systematic review aimed to critically synthesize the available clinical evidence on TPS in PD and identify priorities for future therapeutic and mechanistic research.

## 2 Methods

This systematic review was conducted in accordance with the Preferred Reporting Items for Systematic Reviews and Meta‐Analyses (PRISMA 2020) statement and aimed to synthesize the available evidence regarding the clinical, neurophysiological, neuroimaging, and safety effects of TPS in individuals with PD. The review protocol was prospectively registered in the International Prospective Register of Systematic Reviews (PROSPERO CRD420261459735).

### 2.1 Eligibility Criteria

Studies were eligible if they included adults (≥18 years) with idiopathic PD and investigated TPS as a therapeutic or experimental neuromodulation intervention. Participants were eligible regardless of sex, disease duration, Hoehn and Yahr stage, symptom severity, or medication status. Studies of mixed neurological populations were included only when data for participants with PD could be extracted separately. TPS was defined as the noninvasive application of ultrashort acoustic pulses to targeted brain regions using a dedicated TPS system. Randomized controlled trials, randomized crossover trials, non‐randomized controlled studies, prospective or retrospective interventional studies, and single‐arm clinical studies were eligible if they reported at least one motor, non‐motor, cognitive, neurophysiological, neuroimaging, feasibility, or safety outcome.

Studies were excluded if they involved animal or in vitro models, pediatric populations, healthy participants without separately reported PD data, or patients with secondary, drug‐induced, vascular, or atypical parkinsonism unless data for idiopathic PD were reported separately. Case reports, reviews, editorials, study protocols, trial registrations, conference abstracts without sufficiently detailed or extractable results, and short‐form letters or correspondence that did not constitute a full‐length original clinical report with sufficient methodological information for design‐specific appraisal were excluded from the primary evidence synthesis.

Only studies explicitly investigating TPS were included. Studies evaluating other forms of transcranial ultrasound stimulation, including transcranial focused ultrasound or low‐intensity focused ultrasound not specifically identified as TPS, were excluded. High‐intensity or ablative magnetic resonance‐guided focused ultrasound procedures, including thalamotomy, subthalamotomy, or pallidotomy, as well as ultrasound procedures primarily intended for blood–brain barrier opening, drug or gene delivery, or thermal lesioning, were also excluded. Detailed eligibility criteria and operational definitions are provided in Table S1A.

### 2.2 Information Sources and Search Strategy

A comprehensive literature search was conducted in PubMed/MEDLINE, Embase, and Scopus from database inception to July 26, 2026. The final database search was performed on July 26, 2026. Search strategies combined controlled vocabulary, where applicable, with free‐text terms related to PD, TPS, transcranial acoustic pulse stimulation, ultrasound neuromodulation, transcranial ultrasound, focused ultrasound, and low‐intensity focused ultrasound.

To complement the electronic database search, backward citation searching was performed by manually screening the reference lists of all included studies and relevant reviews. Forward citation searching was performed in Scopus using the “Cited by” function for each included study and relevant TPS review. These supplementary citation searches were completed on July 26, 2026, and identified three additional reports.

Clinical trial registries, including ClinicalTrials.gov and the WHO International Clinical Trials Registry Platform, were not systematically searched. The review was designed to synthesize completed clinical studies with sufficient published data for assessment of clinical, neurophysiological, neuroimaging, and safety outcomes. Trial registrations and study protocols without completed and extractable outcome data were therefore not eligible for the primary evidence synthesis. This is acknowledged as a limitation because ongoing or unpublished studies may not have been captured. The complete electronic search strategies for all databases are provided in Table S1B.

### 2.3 Study Selection

Following duplicate removal, two reviewers independently screened the titles and abstracts of all retrieved records according to the predefined eligibility criteria. Reports considered potentially eligible by either reviewer were retrieved for full‐text assessment. The same reviewers independently evaluated the full‐text reports and documented the primary reason for exclusion of each ineligible report.

Multiple publications arising from the same study population were linked and assessed together. When a conference abstract, preprint, and peer‐reviewed publication described the same study, the peer‐reviewed report containing the most complete data was selected as the primary publication. Companion publications were retained only when they reported distinct eligible outcomes that were not available in the primary report.

Disagreements at either stage were resolved through discussion and consensus, with consultation of a third reviewer when necessary. The complete study‐selection process was documented using a PRISMA 2020 flow diagram (Figure 1).

**FIGURE 1:**
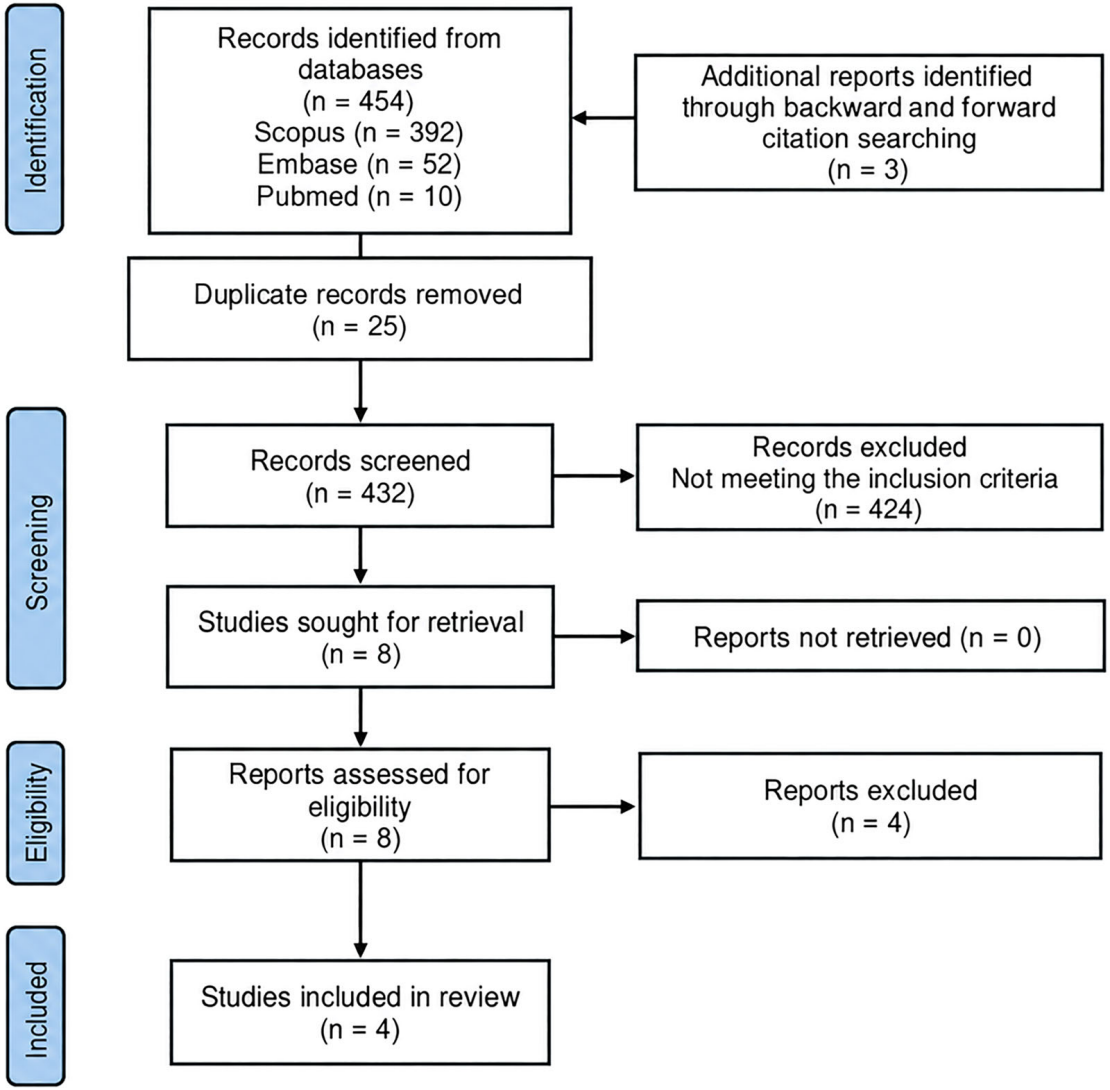
PRISMA 2020 flow diagram of the study‐selection process.

### 2.4 Data Extraction

Two reviewers (G.S.P. and M.L.S.) independently extracted data using a standardized form, with disagreements resolved by consensus or consultation with a third reviewer (J.E.A.). Extracted information included study design, participant characteristics, PD severity and medication state, TPS device and stimulation protocol, comparator, follow‐up, clinical and biological outcomes, adverse events, and principal findings.

For controlled studies, direct active‐versus‐comparator estimates were prioritized over within‐group changes whenever available. Numerical results, effect estimates, confidence intervals, and measures of statistical uncertainty were extracted as reported in the original publications. The complete data‐extraction framework and variables collected are provided in Table S1C.

### 2.5 RoB Assessment

The methodological quality of randomized controlled and randomized crossover trials was assessed using the Cochrane RoB 2 tool. RoB was evaluated across the domains of bias arising from the randomization process, bias due to deviations from intended interventions, bias due to missing outcome data, bias in measurement of outcomes, and bias in selection of the reported result. For crossover trials, the RoB 2 considerations specific to crossover designs were applied, including potential period and carryover effects.

Each randomized study was classified as presenting low RoB, some concerns, or high RoB according to the RoB 2 framework. Non‐randomized and uncontrolled clinical studies were assessed using the ROBINS‐I tool corresponding to the respective study design. Non‐randomized studies were assessed using the ROBINS‐I tool across seven domains: bias due to confounding, bias in selection of participants, bias in classification of interventions, bias due to deviations from intended interventions, bias due to missing data, bias in measurement of outcomes, and bias in selection of the reported result. Overall judgments were classified as low, moderate, serious, critical RoB, or no information, according to ROBINS‐I guidance.

Two reviewers (G.S.P. and M.L.S.) performed the assessments independently. Disagreements were resolved through discussion and consensus or consultation with a third reviewer (J.E.A.).

When information required for RoB assessment was insufficiently reported, clarification was sought from the corresponding authors.

### 2.6 Certainty of Evidence

The certainty of the evidence was assessed using the Grading of Recommendations Assessment, Development, and Evaluation (GRADE) approach. The certainty of evidence was assessed separately according to study design. Evidence from randomized sham‐controlled trials and evidence from non‐randomized or uncontrolled studies were not combined into a single GRADE judgment. Randomized evidence initially received a high‐certainty rating, whereas non‐randomized evidence initially received a low‐certainty rating, with subsequent downgrading according to RoB, inconsistency, indirectness, and imprecision. Uncontrolled studies were interpreted as supportive evidence and not as confirmatory evidence of efficacy.

### 2.7 Data Synthesis

Because substantial clinical and methodological heterogeneity was identified across the included studies with respect to study design, participant characteristics, PD severity, medication status, TPS targets, treatment schedules, number of sessions, acoustic parameters, comparator conditions, follow‐up periods, outcome measures, and reported effect estimates, a quantitative meta‐analysis was not considered appropriate. Therefore, the findings were synthesized narratively in accordance with the Synthesis Without Meta‐analysis reporting guideline.

The results were organized according to four principal domains: clinical motor outcomes; non‐motor, cognitive, and quality‐of‐life outcomes; neurophysiological and neuroimaging outcomes; and safety and tolerability. Studies were additionally grouped according to design, comparator, stimulation target, single‐session or repeated‐session protocol, and timing of outcome assessment.

Whenever possible, findings from active TPS were compared with sham stimulation or baseline values. The magnitude, direction, statistical significance, and durability of the reported effects were considered. Neurophysiological and neuroimaging findings were interpreted alongside clinical outcomes to determine whether TPS‐related changes in cortical oscillations, excitability, functional activation, connectivity, or structural integrity accompanied improvements in motor or non‐motor performance.

Given the small number of studies and the inclusion of uncontrolled investigations, conclusions were based on the consistency and direction of findings rather than vote counting based solely on statistical significance. Particular attention was given to placebo effects, practice effects, small sample sizes, incomplete safety reporting, and the distinction between exploratory mechanistic findings and clinically meaningful treatment effects.

## 3 Results

### 3.1 Study Selection

The database search identified 454 records, including 392 from Scopus, 52 from Embase, and 10 from PubMed/MEDLINE. After removal of 25 duplicate records, 429 unique database records remained for title and abstract screening. Backward and forward citation searching identified three additional reports, resulting in 432 records screened.

After title and abstract screening, 424 records were excluded because they did not meet the predefined eligibility criteria. Eight reports were retrieved and assessed in full text.

Four reports were excluded at the full‐text stage: one study protocol without completed outcome data, one conference abstract superseded by a subsequent peer‐reviewed full‐text publication, one preprint superseded by the final peer‐reviewed publication, and one short‐form correspondence reporting additional uncontrolled clinical observations that did not meet the eligibility criteria for the primary evidence synthesis.

Four clinical studies met all eligibility criteria and were included in the narrative synthesis. The complete study‐selection process is shown in the PRISMA 2020 flow diagram (Figure 1), and the individual full‐text exclusions and specific reasons for exclusion are provided in Table S2.

### 3.2 Characteristics of Included Studies

The four included studies were published between 2024 and 2026 and enrolled a total of 80 adults with idiopathic PD. Sample sizes ranged from 14 to 30 participants. Two studies were conducted in Austria, one in Italy, and one in the United States.

The methodological designs varied considerably. Osou et al. (2024) conducted a retrospective, open‐label, uncontrolled analysis of clinical data from 20 participants. Manganotti et al. (2025) evaluated the effects of a single TPS session in 16 participants with tremor‐dominant PD, 9 of whom additionally underwent the sham condition. Gianlorenco et al. (2026) conducted a prospective, open‐label, single‐arm pilot study involving 14 participants. Matt et al. (2026) performed a randomized, double‐blind, sham‐controlled crossover trial involving 30 participants with mild PD.

TPS protocols ranged from a single stimulation session to 12 sessions administered over 4 weeks. Osou et al. (2024) administered 10 sessions over 2 weeks using primarily sensorimotor, supplementary motor, and cingulate motor targets. Manganotti et al. (2025) administered a single MRI‐navigated session targeting M1 contralateral to the upper limb most affected by resting tremor. Gianlorenco et al. (2026) administered 12 sessions over 4 weeks using individualized cortical, holocranial, and plantar stimulation. Matt et al. (2026) administered six verum and six sham sessions in a crossover design targeting bilateral primary motor area (M1), primary somatosensory cortex (S1), and supplementary motor area (SMA). Clinical outcomes included Unified Parkinson's Disease Rating Scale (UPDRS)‐based motor scores, manual dexterity, accelerometer‐derived resting‐tremor amplitude, non‐motor symptom burden, cognition, depression, and quality of life. Neurobiological outcomes included electroencephalography (EEG), task‐based fMRI, and diffusion tensor imaging (DTI). Follow‐up ranged from immediately after a single TPS session to 1 month after repeated treatment. The main study characteristics and findings are summarized in Table 1.

**TABLE 1:** Characteristics and principal findings of the included studies.

Study	Participants and design	Disease severity and medication state	TPS target and protocol	Comparator and follow‐up	Outcomes	Principal findings	Safety
Osou et al. (2024), Austria	Retrospective, open‐label, uncontrolled clinical study; *n* = 20; 15 men/5 women; mean age 67.6 ± 7.5 years; mean disease duration 53.5 ± 28.0 months	Heterogeneous PD subtypes and comorbidities. Participants remained on optimized standard treatment. Clinical assessments were performed in the ON‐medication state	NEUROLITH TPS; 10 sessions over 2 weeks, generally one session per weekday; approximately 30–45 min/session. Standard protocol: 4000 pulses/session, 4 Hz, 0.25 mJ/mm^2^, pulse duration approximately 3 µs. MRI‐guided individualized targeting of the primary sensorimotor cortex, SMA, and cingulate motor area, with additional symptom‐related targets when indicated. Individual adjustments were permitted: Nine participants received a 50% pulse‐dose reduction during the first session, and one received reduced energy for comfort	No concurrent control group. Assessments occurred within 4 weeks before and after treatment, on average 14 days before the first and 13 days after the final session	UPDRS‐III; adverse events; perceived pressure; and pain	UPDRS‐III decreased from 16.70 ± 8.85 to 12.95 ± 8.55 (within‐group *p* < 0.001; Cohen's *d* = 1.38). A total of 19 of 20 participants improved, and 7 showed reductions of ≥5 points. Because no comparator was included, treatment‐specific effects cannot be distinguished from placebo, temporal variability, or other nonspecific influences	No serious adverse events. Thirteen participants reported ≥1 mild event: fatigue (*n* = 10), headache (*n* = 6), and dizziness (*n* = 6). All resolved within 1 day
Manganotti et al. (2025), Italy	Exploratory single‐session clinical study: *n* = 16 received active TPS (11 men/5 women; age 52–83 years); 9 participants additionally underwent sham TPS in a within‐participant comparison	Idiopathic tremor‐dominant PD. Interventions and assessments were conducted in the ON‐medication state	NEUROLITH TPS: one approximately 30‐min MRI‐navigated session targeting M1 contralateral to the upper limb most affected by resting tremor; 1500 pulses, 4 Hz, 0.20 mJ/mm^2^, delivered in three blocks of 500 pulses separated by 5‐min intervals	Nine participants underwent active and sham sessions on separate days with ≥30‐day washout. Sham used an approximately 3‐cm plastic separator to prevent effective cortical stimulation. Assessments at baseline, immediately after stimulation, and 24 h later	Accelerometer‐derived resting‐tremor amplitude and frequency; UPDRS‐III; UPDRS‐III tremor subscore; subjective tremor, rigidity, and bradykinesia	In the full active cohort, tremor amplitude decreased from 615.2 ± 521.0 µV at baseline to 343.2 ± 307.0 µV immediately after TPS and 347.8 ± 343.2 µV at 24 h. In the sham‐controlled subsample, the immediate reduction was greater after active TPS than sham (*p* = 0.004; *d* = 1.341). Improvement relative to the active‐treatment baseline remained significant at 24 h, whereas direct active‐vs.‐sham superiority was not clearly established at 24 h. Tremor frequency did not change. UPDRS‐III decreased within the full active cohort from 28.8 ± 14.6 to 22.6 ± 13.6 at 24 h	No side effects were reported
Gianlorenco et al. (2026), United States	Prospective, open‐label, single‐arm pilot study; *n* = 14; 10 men/4 women; mean age 69.36 ± 8.53 years. Conducted at Spaulding Rehabilitation Hospital/Mass General Brigham, Massachusetts	Hoehn and Yahr stages 2–4. Stable dopaminergic medication for ≥30 days; mean levodopa‐equivalent daily dose 681.79 ± 644 mg. Assessments were performed in the ON‐medication state	NEUROLITH TPS; 12 sessions over 4 weeks, 3 sessions/week; approximately 50 min/session. 10,000 pulses/session: 8000 transcranial pulses at 4 Hz and 0.25 mJ/mm^2^ plus 2000 plantar pulses at 4 Hz and 0.10 mJ/mm^2^. Transcranial stimulation comprised 4000 individualized symptom‐targeted pulses, including bilateral M1 and selected DLPFC, inferior frontal, posterior parietal, or orbitofrontal regions, plus 4000 standardized holocranial pulses	No control group. Assessments at baseline, after sessions 3, 6, 9, and 12, and at 1‐month follow‐up	UPDRS total and Parts I–IV; NMSS; SCOPA‐COG; PDQ‐39 SI; BDI‐II; PDSS‐2; PFS‐16; FOGQ; TUG; sensory and voice outcomes; resting‐state EEG; safety, feasibility, and acceptability	At session 12, UPDRS total decreased by 9.43 points (*p* < 0.001; *d* = −0.566) and UPDRS‐III by 4.93 points (*p* < 0.001; *d* = −0.498). NMSS decreased by 19.14 points, SCOPA‐COG increased by 7.28 points, PDQ‐39 SI decreased by 1.30 points, and BDI‐II decreased by 3.43 points. Improvements remained detectable at 1 month. EEG showed increases in relative theta activity and theta/alpha and theta/beta ratios. Interpretation is limited by the uncontrolled, multicomponent protocol	Across 168 sessions, three transient mild stimulation‐related events occurred: two episodes of mild pain/pressure and one mild headache. All resolved before participants left the facility. No serious adverse events were reported
Matt et al. (2026), Austria	Randomized, double‐blind, sham‐controlled crossover trial; *n* = 30 randomized; 22 men/8 women; mean age 66.87 ± 7.03 years; 28 completed both treatment cycles	Mild PD; Hoehn and Yahr stages 1–2.5; mean disease duration 5.30 ± 3.35 years. Dopaminergic medication remained stable	Prototype NEUROLITH TPS; six verum and six sham sessions. Each treatment cycle comprised six sessions over 2 weeks, 3 sessions/week. Each approximately 30‐min session delivered 6000 pulses at 4 Hz and 0.25 mJ/mm^2^, pulse duration approximately 3 µs, to individually MRI‐defined bilateral M1, S1, and SMA regions	Verum–sham or sham–verum sequences, *n* = 15 each; acoustically similar blocking cap for sham; 4‐week washout. Clinical assessments at baseline, approximately 1 day after treatment, and 1 month; MRI at baseline and approximately 1 day posttreatment	Co‐primary: MDS‐UPDRS‐III and coin rotation. Secondary: MDS‐UPDRS‐II, B‐ADL, cognition, depression, leisure behavior, task‐based fMRI, DTI, and safety	Left‐hand coin rotation showed a significant Condition × Session interaction (*p* = 0.024; *ηp* ^2^ = 0.14), with significant verum–sham differences immediately after treatment and at 1 month (both corrected *p* = 0.048). No corresponding right‐hand benefit was demonstrated. MDS‐UPDRS‐III improved over time but similarly after verum and sham (Condition × Session *p* = 0.991). No treatment‐specific effects were found for MoCA, BDI‐II, GDS, or MDS‐UPDRS‐II; patient‐rated B‐ADL improved from verum baseline to posttreatment. Verum TPS increased sensorimotor activation on fMRI and fractional anisotropy in right postcentral white matter. The fMRI task was performed with the right hand and should not be interpreted as a direct neural correlate of the left‐hand behavioral effect	Twenty‐one adverse events were reported, all mild and transient. No serious treatment‐related adverse events or treatment‐related neuropathological MRI abnormalities were observed

Abbreviations: B‐ADL, Bayer activities of daily living scale; BDI‐II, Beck depression inventory‐II; DLPFC, dorsolateral prefrontal cortex; DTI, diffusion tensor imaging; EEG, electroencephalography; fMRI, functional magnetic resonance imaging; FOGQ, Freezing of Gait Questionnaire; GDS, Geriatric Depression Scale; M1, primary motor cortex; MDS‐UPDRS, Movement Disorder Society–sponsored revision of the Unified Parkinson's Disease Rating Scale; MoCA, Montreal Cognitive Assessment; NMSS, Non‐Motor Symptoms Scale; PD, Parkinson's disease; PDQ‐39 SI, Parkinson's Disease Questionnaire‐39 Summary Index; PFS‐16, Parkinson's Disease Fatigue Scale; S1, primary somatosensory cortex; SCOPA‐COG, Scales for Outcomes in Parkinson's Disease–Cognition; SMA, supplementary motor area; TPS, transcranial pulse stimulation; TUG, Timed Up and Go; UPDRS, Unified Parkinson's Disease Rating Scale.

All four studies assessed at least one motor outcome, although the magnitude and persistence of the reported effects differed according to study design, number of sessions, and outcome measure.

Osou et al. (2024) reported a significant reduction in UPDRS Part III scores after 10 TPS sessions. Mean scores decreased from 16.70 ± 8.85 at baseline to 12.95 ± 8.55 after treatment, corresponding to a large within‐group effect size (*p* < 0.001; Cohen's *d* = 1.38). Seven of the 20 participants achieved an improvement of at least five points. However, because the study was retrospective and did not include a sham or untreated comparator, the specific contribution of TPS could not be separated from placebo effects, temporal variation, medication‐related influences, or repeated assessment.

Manganotti et al. (2025) specifically investigated resting tremor. Active TPS produced a greater immediate reduction in accelerometer‐derived resting‐tremor amplitude than sham stimulation. Improvement relative to the active‐treatment baseline remained significant at 24 h; however, direct active‐versus‐sham superiority was not clearly established at that time point. These findings suggest a possible short‐term neuromodulatory effect on tremor but do not establish sustained clinical efficacy.

Gianlorenco et al. (2026) reported progressive motor improvement during a 12‐session treatment course. After the final session, total UPDRS decreased by 9.43 points (*p* < 0.001; Cohen's *d* = −0.566), whereas UPDRS Part III decreased by 4.93 points (*p* < 0.001; *d* = −0.498). The improvements remained detectable at the 1‐month follow‐up. Nevertheless, these findings were derived from a small, open‐label, single‐arm study and therefore require confirmation in a sham‐controlled trial.

Matt et al. (2026) provided the strongest controlled evidence. Manual dexterity, measured using the coin rotation task, improved significantly more after verum TPS than after sham stimulation both immediately after treatment and at the 1‐month follow‐up. The corrected probability value was 0.048 at both time points, with partial eta‐squared values of 0.14 immediately after treatment and 0.17 at 1 month. In contrast, gross motor symptoms assessed using the Movement Disorder Society–sponsored revision of the Unified Parkinson's Disease Rating Scale (MDS‐UPDRS) Part III improved after both verum and sham TPS, without evidence of a specific advantage of active treatment. Thus, the controlled findings support a domain‐specific effect on manual dexterity rather than a consistent improvement in global motor impairment.

Overall, repeated TPS was associated with improvements in global motor scores in the uncontrolled studies, whereas the randomized crossover trial demonstrated a more specific benefit for manual dexterity. Improvement relative to baseline persisted at 24 h after active TPS; however, direct active‐versus‐sham superiority was not clearly established at that time point.

### 3.3 Non‐Motor, Cognitive, and Quality‐of‐Life Outcomes

Non‐motor outcomes were evaluated most comprehensively by Gianlorenco et al. (2026). After 12 TPS sessions, total non‐motor symptom burden decreased by 19.14 points (*p* = 0.014; Cohen's *d* = −0.754). Cognitive performance assessed using Scales for Outcomes in Parkinson's Disease–Cognition (SCOPA‐COG) improved by 7.28 points (*p* < 0.001; *d* = 1.161). Parkinson's Disease Questionnaire‐39 Summary Index (PDQ‐39 SI) decreased by 1.30 points (*p* = 0.005; *d* = −0.690), whereas Beck depression inventory‐II (BDI‐II) decreased by 3.43 points (*p* = 0.008; *d* = −0.644).

The same study reported improvements in quality of life and depressive symptoms. The PDQ‐39 SI improved by 1.30 points (*p* = 0.005; *d* = −0.690), whereas the BDI‐II score decreased by 3.43 points (*p* = 0.008; *d* = −0.644). These effects remained detectable 1 month after the end of treatment.

Matt et al. (2026) also evaluated cognition, depressive symptoms, and activities of daily living. No significant Condition × Session interaction was observed for Montreal Cognitive Assessment (MoCA) (*p* = 0.837, *ηp* ^2^ = 0.01), BDI‐II (*p* = 0.361, *ηp* ^2^ = 0.03), Geriatric Depression Scale (GDS) (*p* = 0.414), self‐rated MDS‐UPDRS‐II (*p* = 0.791, *ηp* ^2^ = 0.01), externally rated MDS‐UPDRS‐II (*p* = 0.476, *ηp* ^2^ = 0.02), or leisure‐behavior measures. Patient‐rated Bayer activities of daily living scale (B‐ADL) scores showed a significant overall change (*p* = 0.004), with improvement from the verum baseline to poststimulation assessment (*p* = 0.028); however, externally rated B‐ADL did not show a significant treatment‐specific effect.

Although these results suggest that TPS may influence motor and non‐motor domains, evidence for cognition, mood, and quality of life was derived from a single uncontrolled pilot study. Consequently, the findings may have been influenced by expectancy effects, repeated testing, regression to the mean, or concomitant treatment.

### 3.4 Neurophysiological and Neuroimaging Outcomes

Two studies investigated neurophysiological or neuroimaging changes associated with TPS.

Gianlorenco et al. (2026) reported electroencephalographic changes accompanying the observed clinical improvements. The findings were interpreted as evidence of altered cortical oscillatory activity and enhanced neuroplasticity after repeated TPS. However, because the study lacked a sham comparator, it was not possible to determine whether the EEG changes were specifically attributable to TPS or whether they represented nonspecific longitudinal variation.

Matt et al. (2026) evaluated both functional and structural imaging outcomes. Task‐based fMRI performed during the coin rotation task demonstrated greater activation within the sensorimotor network after verum TPS than after sham stimulation (*p* < 0.001). DTI also indicated increased structural integrity in primary somatosensory white matter after verum TPS, with findings surviving family‐wise error correction (*p* < 0.05). These imaging findings provide complementary evidence of sensorimotor target engagement but should not be interpreted as direct neural correlates of the left‐hand dexterity improvement, because the fMRI coin‐rotation task was performed with the right hand.

Taken together, the available studies suggest that TPS may produce measurable changes in cortical oscillations, task‐related sensorimotor activation, and white‐matter microstructure. Nevertheless, these outcomes were reported in only two small studies using different techniques and analytical approaches. They should therefore be regarded as exploratory mechanistic findings rather than validated biomarkers of treatment response.

### 3.5 Safety and Tolerability

Safety outcomes were reported across all four included studies, although the level of detail varied. Osou et al. (2024) reported no serious adverse events. Mild and transient events occurred in 13 of 20 participants, including fatigue (*n* = 10), headache (*n* = 6), and dizziness (*n* = 6); all resolved within 1 day.

Manganotti et al. (2025) reported no treatment‐related side effects following either active or sham stimulation.

Gianlorenco et al. (2026) reported three mild and transient stimulation‐related adverse events across 168 TPS sessions: two episodes of local pain or pressure and one mild headache. All events resolved before participants left the study site, and no serious adverse events occurred.

Matt et al. (2026) reported 21 adverse events during the crossover trial, all of which were mild and transient. No serious treatment‐related adverse events or treatment‐related neuropathological MRI abnormalities were observed.

Overall, the available evidence suggests favorable short‐term tolerability of TPS. However, the small cumulative sample size, short follow‐up periods, and variable adverse‐event ascertainment limit conclusions regarding uncommon, delayed, or cumulative adverse effects.

### 3.6 Risk of Bias

The RoB was presented according to study design and is summarized in Figure 2.

**FIGURE 2:**
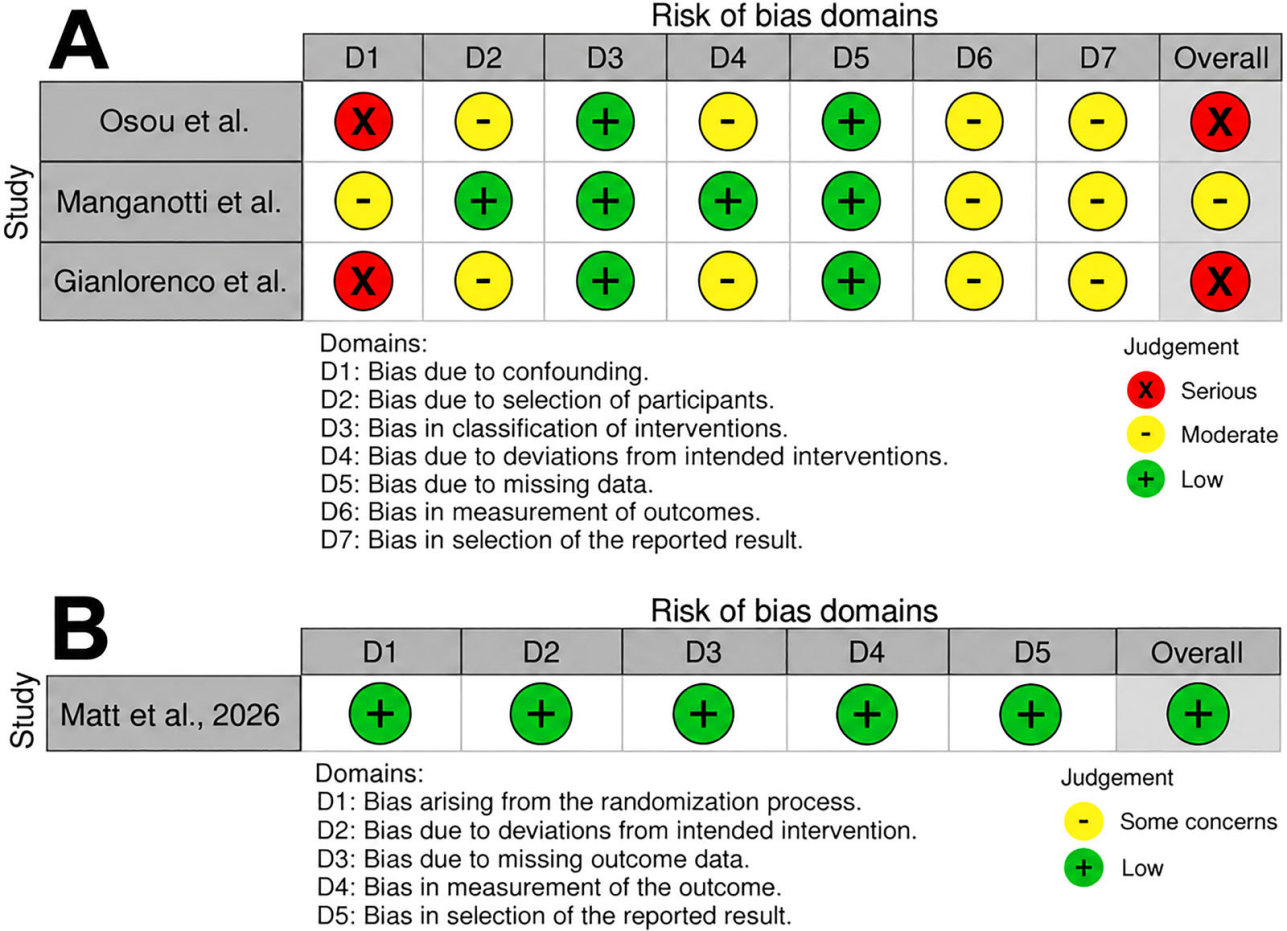
Risk‐of‐bias assessment of the included clinical studies. (A) Risk‐of‐bias assessment of randomized controlled studies using the Cochrane Risk of Bias 2 (RoB 2) tool. (B) Risk‐of‐bias assessment of non‐randomized studies using the Risk Of Bias In Non‐randomized Studies of Interventions (ROBINS‐I) tool. Domain‐level and overall risk‐of‐bias judgments are presented for each study according to the corresponding assessment framework.

Among the non‐randomized studies, Osou et al. (2024) presented serious concerns related primarily to confounding, the retrospective uncontrolled design, and potential bias in outcome measurement and reporting. Manganotti et al. (2025) included a sham condition in a small within‐participant subsample, but limited reporting of treatment sequence, allocation procedures, masking, and the selection of participants contributing to the sham comparison reduced confidence in the findings. Gianlorenco et al. (2026) was prospective but open‐label and uncontrolled, leaving the study vulnerable to confounding, placebo effects, regression to the mean, repeated testing, and observer‐related bias.

The randomized crossover trial by Matt et al. (2026) showed several methodological strengths. The principal investigator and the neuropsychological and radiological evaluators were blinded, whereas the TPS operator was aware of the treatment condition but had no role in neuropsychological assessment or data analysis. The randomization‐process domain was judged to be at low RoB. No material concerns were identified in the remaining domains, resulting in an overall low‐RoB judgment for the trial.

Overall, the evidence base remained methodologically limited. Although the randomized trial was judged to be at low RoB, the non‐randomized and uncontrolled studies were still particularly vulnerable to confounding, placebo effects, regression to the mean, and other nonspecific influences.

### 3.7 Certainty of Evidence

The certainty of evidence varied according to study design and outcome domain (Table 2). For global motor function, randomized evidence was rated as moderate certainty, with one‐level downgrading for imprecision because the evidence was derived from a single small crossover trial. The controlled analysis did not demonstrate a treatment‐specific effect on MDS‐UPDRS Part III. In contrast, favorable UPDRS‐based motor changes reported in non‐randomized and uncontrolled studies were rated as very low certainty because of serious RoB, heterogeneity, and imprecision.

**TABLE 2:** GRADE assessment of the certainty of evidence for transcranial pulse stimulation in Parkinson's disease.

Outcome domain	Evidence/participants	Main findings	Risk of bias	Inconsistency	Indirectness	Imprecision	Publication bias	Certainty	Explanation
Global motor function—randomized evidence	Matt et al. (2026); randomized sham‐controlled crossover trial; *n* = 30	MDS‐UPDRS‐III improved across sessions, but there was no treatment‐specific effect (Condition × Session *p* = 0.991)	Not serious	Not assessable	Not serious	Serious	Not assessed^a^	⨁⨁⨁◯ Moderate	Evidence derives from one small crossover trial. Risk of bias was judged not serious; imprecision remains the principal limitation. The controlled result does not support a verum‐specific effect on global motor impairment
Global motor function—non‐randomized/uncontrolled evidence	Osou et al. (2024), Manganotti et al. (2025), Gianlorenco et al. (2026); *n* = 50	Favorable within‐group reductions in UPDRS‐based motor scores were reported, but these estimates were not supported by adequate concurrent randomized comparisons	Very serious	Serious	Not serious	Serious	Not assessed^a^	⨁◯◯◯ Very low	Evidence is dominated by uncontrolled or exploratory analyses vulnerable to confounding, placebo effects, regression to the mean, and repeated assessment; protocols and outcomes were heterogeneous, and samples were small
Manual dexterity—randomized evidence	Matt et al. (2026); *n* = 30	Verum TPS improved left‐hand coin rotation more than sham immediately after treatment and at 1 month; no corresponding right‐hand benefit was demonstrated	Not serious	Not assessable	Not serious	Serious	Not assessed^a^	⨁⨁⨁◯ Moderate	Single small randomized crossover trial with no independent replication; effect was lateralized and therefore should not be generalized to overall upper‐limb function
Resting tremor—non‐randomized sham comparison	Manganotti et al. (2025); *n* = 16 active; *n* = 9 sham‐controlled subsample	Active TPS reduced resting‐tremor amplitude more than sham immediately after stimulation (*p* = 0.004; *d* = 1.341). Improvement relative to the active baseline persisted at 24 h, but direct active‐vs.‐sham superiority was not clearly established at 24 h	Serious	Not assessable	Not serious	Very serious	Not assessed^a^	⨁◯◯◯ Very low	Only nine participants contributed to the sham comparison, with limited reporting of treatment order and masking and substantial imprecision
Non‐motor, cognitive, and daily‐functioning outcomes—randomized evidence	Matt et al. (2026); *n* = 30	No significant treatment‐specific interactions were demonstrated for MoCA, BDI‐II, GDS, MDS‐UPDRS‐II, or leisure behavior. Patient‐rated B‐ADL improved from verum baseline to posttreatment, but this was not accompanied by consistent controlled benefits across domains	Not serious	Not assessable	Not serious	Serious	Not assessed^a^	⨁⨁⨁◯ Moderate	Controlled evidence is limited to one small crossover trial and does not demonstrate consistent verum‐specific non‐motor benefit
Non‐motor, cognitive, mood, and quality‐of‐life outcomes—uncontrolled evidence	Gianlorenco et al. (2026); *n* = 14	Improvements were reported for NMSS, SCOPA‐COG, BDI‐II, PDQ‐39 SI, and other domains after repeated TPS and remained detectable at 1 month	Very serious	Not assessable	Not serious	Very serious	Not assessed^a^	⨁◯◯◯ Very low	Single open‐label uncontrolled study with a multicomponent intervention and multiple outcomes; placebo effects, repeated testing, confounding, and multiplicity cannot be excluded
EEG cortical oscillatory changes—uncontrolled evidence	Gianlorenco et al. (2026); *n* = 14	Relative theta activity and theta/alpha and theta/beta ratios increased following treatment	Very serious	Not assessable	Not serious^b^	Very serious	Not assessed^a^	⨁◯◯◯ Very low	Evidence of biological change comes from one small uncontrolled study without sham comparison. The clinical meaning and directionality of the EEG changes remain uncertain
Task‐based fMRI target engagement—randomized evidence	Matt et al. (2026); *n* = 28 MRI dataset	Verum TPS increased task‐related sensorimotor activation relative to sham, including right M1 and S1	Not serious	Not assessable	Not serious^b^	Serious	Not assessed^a^	⨁⨁⨁◯ Moderate	Sham‐controlled imaging provides evidence of biological target engagement, but the sample was small, and findings lack independent replication. The fMRI task involved the right hand and was not a direct neural correlate of the left‐hand dexterity benefit
DTI white‐matter changes—randomized evidence	Matt et al. (2026); *n* = 26 DTI dataset	Verum TPS increased fractional anisotropy in right postcentral/sensorimotor white matter relative to sham (*p* < 0.05, FWE‐corrected)	Not serious	Not assessable	Not serious^b^	Serious	Not assessed^a^	⨁⨁⨁◯ Moderate	Controlled structural imaging supports a biological effect but is based on a single small trial with reduced MRI sample size and no independent replication
Safety and tolerability—randomized evidence	Matt et al. (2026); *n* = 30	Twenty‐one adverse events were reported; all were mild and transient. No serious treatment‐related adverse events or treatment‐related neuropathological MRI abnormalities were observed	Not serious	Not assessable	Not serious	Very serious	Not assessed^a^	⨁⨁◯◯ Low	Randomized evidence supports short‐term tolerability and was judged at low risk of bias, but only 30 participants and short follow‐up result in very serious imprecision for uncommon or delayed adverse events
Safety and tolerability—non‐randomized evidence	Osou et al. (2024), Manganotti et al. (2025), Gianlorenco et al. (2026); *n* = 50	No serious treatment‐related adverse events were reported. Reported events were mild and transient; Manganotti et al. reported no side effects	Serious	Not serious	Not serious	Serious	Not assessed^a^	⨁◯◯◯ Very low	Safety findings were generally consistent, but evidence arose from small non‐randomized studies with short follow‐up and variable adverse‐event ascertainment, limiting detection of uncommon, delayed, or cumulative harms

*Note*: Certainty of evidence was assessed using the Grading of Recommendations Assessment, Development, and Evaluation (GRADE) framework. The symbols indicate the following certainty levels: ⨁⨁⨁⨁ = high certainty; ⨁⨁⨁◯ = moderate certainty; ⨁⨁◯◯ = low certainty; and ⨁◯◯◯ = very low certainty. These symbols correspond to the certainty ratings presented in the "Certainty" column of Table 2.

Abbreviations: B‐ADL, Bayer activities of daily living scale; BDI‐II, Beck depression inventory‐II; DTI, diffusion tensor imaging; EEG, electroencephalography; fMRI, functional magnetic resonance imaging; FWE, family‐wise error; GDS, Geriatric Depression Scale; GRADE, Grading of Recommendations Assessment, Development, and Evaluation; MDS‐UPDRS, Movement Disorder Society–sponsored revision of the Unified Parkinson's Disease Rating Scale; MoCA, Montreal Cognitive Assessment; NMSS, Non‐Motor Symptoms Scale; PDQ‐39 SI, Parkinson's Disease Questionnaire‐39 Summary Index; SCOPA‐COG, Scales for Outcomes in Parkinson's Disease–Cognition; TPS, transcranial pulse stimulation; UPDRS, Unified Parkinson's Disease Rating Scale.

^a^ Publication bias was not formally assessed because too few studies contributed to each outcome‐specific evidence stream. No downgrade was applied solely because formal assessment was not possible.

^b^ For EEG, fMRI, and DTI outcomes, indirectness was judged relative to the question of biological target engagement, not clinical therapeutic benefit. These certainty ratings therefore indicate confidence that TPS was associated with the specified biological change and must not be interpreted as certainty that the biomarker change produces a clinically meaningful benefit.

Evidence for manual dexterity was rated as moderate certainty. The randomized crossover trial demonstrated a verum‐specific improvement in left‐hand coin rotation, but the evidence was downgraded for imprecision because it was based on a single trial with 30 participants and lacked independent replication. Evidence for resting tremor was rated as very low certainty because only a small sham‐controlled subsample contributed to the direct comparison, with important methodological limitations and substantial imprecision.

For non‐motor, cognitive, and daily‐functioning outcomes, randomized evidence was rated as moderate certainty. No consistent treatment‐specific benefits were demonstrated across cognition, mood, or activities of daily living. Evidence from the uncontrolled study reporting multidomain improvements was rated as very low certainty because of the single‐arm design, multicomponent intervention, multiple outcomes, and small sample size.

Neurophysiological and neuroimaging outcomes were graded separately. EEG findings were rated as very low certainty, whereas task‐based fMRI and DTI findings from the randomized sham‐controlled trial were rated as moderate certainty, with downgrading for imprecision. These ratings refer specifically to evidence of biological target engagement and should not be interpreted as certainty of clinical therapeutic benefit.

For safety and tolerability, randomized evidence was rated as low certainty because the small sample size and short follow‐up provided limited ability to detect uncommon, delayed, or cumulative adverse events. Non‐randomized evidence was rated as very low certainty. Across both evidence streams, no serious treatment‐related adverse events were reported, and reported adverse events were generally mild and transient. Publication bias could not be formally assessed because too few studies contributed to each outcome‐specific evidence stream.

## 4 Discussion

Four clinical studies involving 80 participants were included in this systematic review (Osou et al. 2024; Manganotti et al. 2025; Gianlorenco et al. 2026; Matt et al. 2026). The two uncontrolled repeated‐session studies reported improvements in global motor outcomes (Osou et al. 2024; Gianlorenco et al. 2026), whereas the randomized sham‐controlled crossover trial did not demonstrate a treatment‐specific effect on MDS‐UPDRS Part III (Matt et al. 2026). The clearest sham‐controlled clinical signal was an improvement in left‐hand manual dexterity after repeated TPS, sustained at 1 month (Matt et al. 2026). A separate single‐session study demonstrated an immediate reduction in resting‐tremor amplitude relative to sham, with improvement relative to the active‐treatment baseline still present after 24 h but without clear active‐versus‐sham superiority at that later assessment (Manganotti et al. 2025). EEG, fMRI, and DTI findings provided preliminary evidence of biological target engagement (Gianlorenco et al. 2026; Matt et al. 2026), and no serious treatment‐related adverse events were reported (Osou et al. 2024; Manganotti et al. 2025; Gianlorenco et al. 2026; Matt et al. 2026). Taken together, current evidence is stronger for selected domain‐specific effects and target engagement than for a generalized clinical benefit of TPS in PD.

Interpretation of global motor changes requires particular caution because the most favorable UPDRS findings were obtained in uncontrolled studies. Osou et al. (2024) and Gianlorenco et al. (2026) reported clinically favorable pre–postreductions, but such changes cannot isolate TPS effects from expectancy, regression to the mean, repeated assessment, medication‐related variation, or natural symptom fluctuation. Placebo responses are particularly relevant in PD because expectation itself can induce striatal dopamine release (de la Fuente‐Fernández et al. 2001). Clinical relevance should also be judged using the appropriate instrument. For the original UPDRS motor scale, a change of approximately five points has been proposed as clinically important in early PD (Schrag et al. 2006), whereas an improvement of approximately 3.25 points has been proposed for MDS‐UPDRS Part III (Horváth et al. 2015). These thresholds should not be used interchangeably, particularly when studies employ different or harmonized versions of the scales. Importantly, in Matt et al. (2026), MDS‐UPDRS Part III improved over time but did so similarly following verum and sham TPS, with no Condition × Session effect, arguing against a specific effect on global motor impairment.

Manual dexterity currently provides the strongest controlled signal for TPS. Matt et al. (2026) demonstrated a significant Condition × Session interaction for left‐hand coin rotation, with improvements after verum relative to sham both immediately after treatment and at 1 month. This finding is potentially relevant because fine hand function contributes directly to daily activities and may not be adequately captured by a broad motor composite. However, the laterality of the findings requires careful interpretation. The significant behavioral benefit concerned the left hand, whereas the fMRI coin‐rotation task was performed with the right hand. Therefore, the fMRI changes should not be described as a direct anatomical correlate of the left‐hand behavioral improvement. Rather, increased sensorimotor activation after verum TPS provides complementary evidence of network‐level target engagement. The DTI finding of increased fractional anisotropy in right postcentral white matter is also consistent with modulation of the stimulated sensorimotor system but remains an exploratory structural biomarker. The behavioral finding arose from a single trial of 30 participants and therefore requires independent replication (Matt et al. 2026).

The tremor findings similarly support a restricted rather than generalized interpretation. Manganotti et al. (2025) showed that active TPS produced a greater immediate reduction in accelerometer‐derived resting‐tremor amplitude than sham. Within the active condition, improvement remained significant relative to baseline at 24 h; however, direct active‐versus‐sham superiority was not clearly demonstrated at this later time point. Tremor frequency was unchanged. This pattern may indicate modulation of the cortical expression or amplitude of tremor without alteration of the fundamental oscillatory frequency generated across the basal ganglia–cerebello‐thalamo‐cortical network. Nevertheless, only 9 of the 16 participants underwent the sham comparison, and the exploratory design limits conclusions regarding persistence and clinical relevance.

Additional preliminary observations were subsequently reported by Manganotti et al. (2026) in a short‐form correspondence describing repeated TPS as an add‐on intervention in PD. Because this publication did not meet the eligibility criteria for inclusion in the primary evidence synthesis, its findings were not incorporated into the formal RoB or GRADE assessments. Nevertheless, the report provides complementary hypothesis‐generating evidence regarding repeated M1 stimulation and reinforces the need for adequately controlled repeated‐session trials.

Non‐motor findings remain less certain. Gianlorenco et al. (2026) reported improvements in non‐motor symptom burden, cognition, depression, sleep, and quality of life following a 12‐session intervention, but the study was open‐label and uncontrolled and combined individualized cortical stimulation, holocranial stimulation, and plantar stimulation. The independent contribution of each component therefore cannot be determined. In contrast, Matt et al. (2026) found no treatment‐specific interaction for MoCA, BDI‐II, GDS, MDS‐UPDRS‐II, or leisure‐behavior outcomes, although patient‐rated B‐ADL improved after verum stimulation. These controlled null findings should be considered when interpreting the multidomain improvements observed in uncontrolled studies. Similarly, the EEG increases in relative theta power, and theta/alpha and theta/beta ratios reported by Gianlorenco et al. (2026) remain difficult to interpret. Increased slow‐frequency activity is not inherently beneficial, and longitudinal PD studies have associated cortical slowing with cognitive decline (Klassen et al. 2011; Olde Dubbelink et al. 2013). The EEG findings therefore provide evidence that cortical dynamics changed over time, but not yet that the observed changes represent a validated therapeutic biomarker.

TPS should also be interpreted within the rapidly expanding literature on other forms of transcranial ultrasound stimulation. TPS and conventional transcranial ultrasound stimulation (TUS)/transcranial focused ultrasound stimulation (tFUS) are not interchangeable: TPS typically uses ultrashort, single‐cycle, broadband pulses at relatively low repetition rates, whereas many contemporary TUS protocols use multi‐cycle sinusoidal bursts at a defined carrier frequency and can be patterned as theta‐burst or other stimulation sequences (Truong et al. 2022). Nevertheless, the broader TUS literature provides important mechanistic context. In an early PD study, accelerated theta‐burst TUS of M1 increased transcranial magnetic stimulation (TMS)‐evoked corticospinal excitability relative to sham without producing a significant change in MDS‐UPDRS Part III, illustrating that physiological target engagement may occur without an immediate clinical effect (Samuel et al. 2023). Subsequent work demonstrated that M1 theta‐burst TUS‐induced plasticity depends on dopaminergic state, with impaired responses to OFF medication and restoration of plasticity to ON medication (Grippe et al. 2024).

More recent TUS studies have extended stimulation to deep motor‐network targets and have reported measurable clinical effects. Bilateral globus pallidus internus (GPi)‐region theta‐burst TUS was associated with reductions in MDS‐UPDRS Part III scores and bradykinesia, whereas M1 stimulation predominantly altered cortical excitability in the same study (Lin et al. 2026). In a randomized crossover study of PD‐related tremor, ventral intermediate nucleus (VIM) stimulation reduced postural tremor, whereas zona incerta stimulation improved both postural and resting tremor and produced imaging changes related to clinical response (Vuong et al. 2026). These observations indicate that target selection may be as important as stimulation modality itself.

The strongest mechanistic advance in the recent TUS literature comes from direct intracranial recordings. Darmani et al. (2025) demonstrated that TUS can modulate local field potentials recorded from implanted GPi electrodes, with stimulation‐pattern‐dependent effects on theta and beta activity. Eraifej et al. (2026) subsequently reported that pallidal TUS reduced ipsilateral subthalamic nucleus (STN) beta power by 10.34% and reduced bradykinesia measured by reaction time by 17.70% in a proof‐of‐concept randomized crossover study. Most recently, Sarica et al. (2026) showed in 17 patients that M1 TUS reduced pathological STN beta oscillations relative to sham and that this modulation was associated with improvement in motor signs; conversely, GPi stimulation using their protocol increased beta activity without comparable motor improvement. The apparently different effects reported across GPi protocols are particularly informative: Ultrasound neuromodulation cannot be understood simply in terms of anatomical target, because pulse pattern, frequency, acoustic exposure, brain state, and network context may determine both the direction and magnitude of the physiological response.

These findings also provide a potential roadmap for TPS development. To date, TPS studies have relied primarily on behavioral measures, EEG, and neuroimaging to infer target engagement (Osou et al. 2024; Manganotti et al. 2025; Gianlorenco et al. 2026; Matt et al. 2026). Future mechanistic studies could combine TPS with local field potential (LFP) recordings in patients with sensing‐enabled deep brain stimulation (DBS) systems to determine whether specific TPS targets or pulse doses suppress pathological beta activity or produce other predefined electrophysiological changes, analogous to approaches recently used with TUS (Darmani et al. 2025; Eraifej et al. 2026; Sarica et al. 2026). Such biomarker‐guided experiments could be used for target selection and dose optimization before larger therapeutic trials. However, LFP‐guided TPS has not yet been demonstrated, and the acoustic waveform of TPS differs substantially from the TUS protocols used in these intracranial studies. It should therefore be viewed as a promising research strategy rather than an established treatment‐optimization approach.

Current evidence does not identify a clear clinical advantage of TPS over other TUS approaches. TPS has potential practical strengths, including a standardized clinical device, MRI‐based neuronavigation, real‐time tracking, and experience with repeated‐session treatment paradigms (Beisteiner et al. 2020; Truong et al. 2022; Matt et al. 2022; Karakatsani, Nozdriukhin, et al. 2025; Osou et al. 2024; Manganotti et al. 2025). Conversely, recent TUS approaches have demonstrated modulation of cortical and deep motor‐network structures, including GPi, VIM, zona incerta, and STN‐connected circuitry, and can be combined with patient‐specific acoustic modeling and intracranial electrophysiology (Lin et al. 2026; Vuong et al. 2026; Darmani et al. 2025; Eraifej et al. 2026; Sarica et al. 2026). No head‐to‐head trial has compared TPS with another TUS waveform in PD. Consequently, claims of greater efficacy, focality, or durability for TPS would currently be premature. The two families of approaches are better regarded as technically distinct and potentially complementary components of ultrasound neuromodulation research.

Several methodological issues should guide future TPS studies. First, protocol heterogeneity remains substantial: stimulation targets ranged from unilateral M1 to bilateral M1/S1/SMA, individualized cortical networks, cingulate areas, holocranial stimulation, and additional peripheral stimulation, with marked differences in cumulative pulse dose and number of sessions (Osou et al. 2024; Manganotti et al. 2025; Gianlorenco et al. 2026; Matt et al. 2026). Second, medication state requires systematic consideration. Dopaminergic therapy can influence cortical plasticity in PD (Morgante et al. 2006), and recent TUS evidence directly demonstrates medication‐dependent neuromodulatory responses (Grippe et al. 2024). Third, future studies should comprehensively report the TPS device, pulse waveform, energy exposure, targeting procedure, coupling conditions, skull‐related attenuation estimates, and estimated intracranial dose. Such reporting is necessary for reproducibility, but reporting frameworks developed for other ultrasound waveforms should not automatically be assumed to apply directly to TPS.

Short‐term tolerability was favorable across the included TPS studies, with no serious treatment‐related adverse events identified (Osou et al. 2024; Manganotti et al. 2025; Gianlorenco et al. 2026; Matt et al. 2026). Reported adverse events were generally mild and transient. Nevertheless, 80 participants are insufficient to characterize uncommon, delayed, or cumulative risks, particularly because follow‐up was short. The evidence base was also constrained by small samples, heterogeneous protocols, and the predominance of uncontrolled designs. These limitations contributed to moderate, low, or very low certainty ratings across outcome domains and precluded a meaningful quantitative meta‐analysis.

This systematic review also has limitations. First, the small number of eligible TPS studies and the substantial heterogeneity in study design, stimulation targets, acoustic parameters, outcome measures, and follow‐up periods precluded quantitative meta‐analysis and limited comparisons across protocols. Second, clinical trial registries were not systematically searched; therefore, ongoing or unpublished TPS studies may not have been identified. Third, the review was restricted to studies explicitly identified as TPS, and findings from other forms of low‐intensity transcranial ultrasound stimulation were not included in the primary evidence synthesis, although they were discussed as mechanistic context. Fourth, several included studies were small, uncontrolled, or exploratory, which limited the certainty of the evidence and prevented robust assessment of publication bias, subgroup effects, and dose‐response relationships. Finally, because TPS is a rapidly evolving field, recently published or forthcoming studies may alter the current evidence base.

Future trials should therefore be prospectively registered, adequately powered, double‐blind, and sham‐controlled, with a clearly defined target, acoustic protocol, primary outcome, and mechanistic hypothesis. Target‐specific behavioral measures should complement global scales such as MDS‐UPDRS Part III. Imaging, EEG, or electrophysiological biomarkers should be prespecified rather than explored across numerous post hoc outcomes, and follow‐up should extend beyond 1 month. Particularly informative designs would examine whether biomarker‐confirmed target engagement predicts subsequent clinical response. At present, the evidence supports the conclusion that TPS can modulate the sensorimotor system and may influence selected outcomes such as manual dexterity and resting‐tremor amplitude; whether these effects can be optimized and translated into consistent, durable clinical benefit remains to be established.

## 5 Conclusion

In conclusion, current evidence does not establish TPS as an effective treatment for global motor impairment, non‐motor symptoms, cognition, or sustained tremor control in PD. The available evidence supports a narrower conclusion: TPS may engage the sensorimotor system and improve selected target‐related outcomes, particularly manual dexterity and short‐term resting tremor amplitude. These findings are biologically plausible and clinically promising, but they require independent confirmation in rigorous sham‐controlled trials before TPS can be recommended for routine clinical use.

## Author Contributions

Palmerindo Antônio Tavares de Mendonça Neto, Gabrielly Santos Pereira, and Gustavo Henrique de Mello Rosa contributed to the literature search, study selection, data extraction, risk‐of‐bias assessment, and drafting of the manuscript. João Eduardo de Araújo and Marcelo Lourenço da Silva contributed to the conception and methodological design of the review, supervision, interpretation of the findings, and critical revision of the manuscript. All authors read and approved the final manuscript.

## Funding

This study was financed in part by the Coordenação de Aperfeiçoamento de Pessoal de Nível Superior—Brasil (CAPES)—Finance Code 001.

## Ethics Statement

The authors have nothing to report.

## Consent

The authors have nothing to report.

## Conflicts of Interest

The authors declare no conflicts of interest.

## Supporting information




**Supplementary Material**: brb371895‐sup‐0001‐TableS1.docx

**Supplementary Material**: brb371895‐sup‐0002‐TableS2.docx

## Data Availability

All data analyzed in this systematic review were obtained from previously published studies cited in the manuscript. Review‐generated materials, including data‐extraction and methodological‐appraisal records, are available from the corresponding author upon reasonable request.
