# Supplementary material for: Transcranial Pulse Stimulation in Parkinson's Disease: A Systematic Review
Source: Brain Behav. 2026 Nov 3;16(11):e71895. doi: 10.1002/brb3.71895 (PMC13729823; doi:10.1002/brb3.71895)
Supplement: Supplementary file 1 [file BRB3-16-e71895-s001.docx]

**Supplementary Table S2. Full-text reports excluded from the primary evidence synthesis and reasons for exclusion**

Eight reports were retrieved and assessed for eligibility. Four reports met the eligibility criteria and were included in the primary narrative synthesis; the four reports below were excluded for the specific reasons shown.

| **Full-text report** | **Publication type** | **Reason for exclusion** |
| --- | --- | --- |
| Gianlorenço AC, Camargo L, Fernandes EB, Pichardo E, Yeh HJ, Hazer-Rau D, Storz R, Fregni F. Study Protocol: Investigating the Effects of Transcranial Pulse Stimulation in Parkinson’s Disease. Bioengineering (Basel). 2025;12(7):773. doi:10.3390/bioengineering12070773. | Study protocol | Protocol report without completed clinical outcome data. The subsequent completed clinical study was assessed separately and included in the review. |
| Manganotti P, Liccari M, Lombardo TMI, Michelutti M, Catalan M. Transcranical Pulse Stimulation in Parkinson’s rest tremor: a pilot study. [abstract]. Mov Disord. 2023;38(Suppl 1). Abstract No. 1223. | Conference abstract | Conference abstract reporting preliminary data from the same clinical research program later published as a full peer-reviewed article. The subsequent publication reported an expanded sample and additional sham-controlled data and was therefore used as the primary report. |
| Matt E, Plischek N, Mitterwallner M, Radjenovic S, Weber A, Domitner A, et al. Transcranial Pulse Stimulation Enhances Dexterity in Parkinson’s Disease: A Randomized Sham-Controlled Clinical Trial. medRxiv. 2025. doi:10.1101/2025.07.07.25330807. | Preprint | Superseded by the final peer-reviewed publication in Movement Disorders, which was used as the primary report. |
| Manganotti P, Liccari M, Lombardo TMI, Della Toffola J, Cenacchi V, Ajčević M, et al. Effects of Transcranial Pulse Stimulation (TPS) as a potential “add-on” intervention in patients with Parkinson’s disease. Parkinsonism Relat Disord. 2026;142:108128. doi:10.1016/j.parkreldis.2025.108128. | Correspondence / Letter | Short-form report of additional uncontrolled clinical observations that did not meet the eligibility criterion requiring sufficient methodological information for complete design-specific appraisal in the primary evidence synthesis. The report was considered as complementary emerging evidence in the Discussion but was not included in the formal risk-of-bias or GRADE analyses. |

**PRISMA accounting:** 8 reports retrieved and assessed for eligibility; 4 full-text reports excluded; 4 clinical studies included in the primary synthesis.
