# Supplementary material for: Transcranial Pulse Stimulation in Parkinson's Disease: A Systematic Review
Source: Brain Behav. 2026 Nov 3;16(11):e71895. doi: 10.1002/brb3.71895 (PMC13729823; doi:10.1002/brb3.71895)
Supplement: Supplementary file 2 [file BRB3-16-e71895-s002.docx]

**Supplementary Table S1. Detailed eligibility criteria, complete electronic search strategies, and data-extraction framework**

**A. Detailed eligibility criteria and operational definitions**

| **Domain** | **Eligibility criterion / operational definition** |
| --- | --- |
| **Population** | Adults aged ≥18 years with idiopathic Parkinson’s disease diagnosed according to recognized clinical criteria or confirmed by a qualified healthcare professional. Participants were eligible regardless of sex, disease duration, Hoehn and Yahr stage, symptom severity, or medication status. Mixed neurological populations were eligible only when Parkinson’s disease data were separately extractable. |
| **Intervention** | Transcranial pulse stimulation (TPS) used as a therapeutic or experimental neuromodulation intervention. TPS was operationally defined as the non-invasive application of ultrashort acoustic pulses to targeted brain regions using a dedicated TPS system. |
| **Eligible TPS protocols** | No restriction was imposed on cortical target, energy flux density, pulse repetition frequency, total pulse number, number of sessions, treatment schedule, neuronavigation method, or follow-up duration. |
| **Comparators** | Sham TPS, no-treatment/usual-care comparators, within-participant control conditions, or no comparator for eligible uncontrolled clinical studies. |
| **Eligible study designs** | Randomized controlled trials, randomized crossover trials, non-randomized controlled studies, prospective or retrospective interventional studies, and single-arm clinical studies. |
| **Motor outcomes** | Validated or quantitative measures of motor function, including UPDRS/MDS-UPDRS motor scores, tremor, bradykinesia, rigidity, gait, balance, manual dexterity, reaction time, and activities of daily living. |
| **Non-motor outcomes** | Cognition, mood or depressive symptoms, sleep, fatigue, non-motor symptom burden, activities of daily living, and health-related quality of life. |
| **Neurophysiological outcomes** | Electroencephalography, cortical oscillatory activity, transcranial magnetic stimulation measures of cortical excitability, motor-evoked potentials, and other electrophysiological biomarkers. |
| **Neuroimaging outcomes** | Task-based or resting-state functional MRI, diffusion tensor imaging, functional connectivity, white-matter indices, and other structural or functional imaging biomarkers. |
| **Safety / feasibility outcomes** | Adverse events, serious adverse events, participant-reported discomfort, treatment discontinuation, adherence, feasibility, and treatment acceptability. |
| **Excluded populations** | Animal or in vitro models, pediatric populations, healthy participants without separately reported Parkinson’s disease data, and secondary, drug-induced, vascular, or atypical parkinsonism unless idiopathic Parkinson’s disease data were reported separately. |
| **Excluded publication types** | Case reports, reviews, editorials, study protocols, trial registrations, conference abstracts without sufficiently detailed and extractable results, and short-form letters/correspondence that did not constitute a full-length original clinical report with sufficient methodological information for design-specific appraisal. |
| **Excluded ultrasound interventions** | Generic transcranial ultrasound stimulation, transcranial focused ultrasound stimulation, or low-intensity focused ultrasound not explicitly described as TPS; high-intensity or ablative MR-guided focused ultrasound (including thalamotomy, subthalamotomy, or pallidotomy); and ultrasound procedures primarily intended for blood–brain barrier opening, drug or gene delivery, or thermal lesioning. |

**B. Complete electronic search strategies**

The databases were searched from inception to 26 July 2026. Broad ultrasound-related terminology was used to maximize sensitivity because terminology in acoustic neuromodulation is not standardized. Records not explicitly investigating TPS were subsequently excluded during screening according to the intervention criteria above.

| **Database / platform** | **Date searched** | **Search strategy** | **Filters / limits** | **Records retrieved** |
| --- | --- | --- | --- | --- |
| PubMed/MEDLINE (PubMed, NLM) | 26 July 2026 | ("Parkinson Disease"[Mesh] OR "Parkinson Disease"[tiab] OR "Parkinson's disease"[tiab] OR parkinson*[tiab]) AND ("transcranial pulse stimulation"[tiab] OR "transcranial acoustic pulse stimulation"[tiab] OR "ultrasound neuromodulation"[tiab] OR "transcranial ultrasound"[tiab] OR "focused ultrasound"[tiab] OR "low-intensity focused ultrasound"[tiab] OR "low intensity focused ultrasound"[tiab] OR tFUS[tiab] OR LIFU[tiab]) | No language, publication-type, or date filters applied. | 10 |
| Embase (Elsevier) | 26 July 2026 | ('parkinson disease'/exp OR 'parkinson disease':ti,ab,kw OR parkinson*:ti,ab,kw) AND ('transcranial pulse stimulation':ti,ab,kw OR 'transcranial acoustic pulse stimulation':ti,ab,kw OR 'ultrasound neuromodulation':ti,ab,kw OR 'transcranial ultrasound':ti,ab,kw OR 'focused ultrasound':ti,ab,kw OR 'low intensity focused ultrasound':ti,ab,kw OR tfus:ti,ab,kw OR lifu:ti,ab,kw) | No language, publication-type, or date filters applied. | 52 |
| Scopus (Elsevier) | 26 July 2026 | TITLE-ABS-KEY(parkinson*) AND TITLE-ABS-KEY("transcranial pulse stimulation" OR "transcranial acoustic pulse stimulation" OR "ultrasound neuromodulation" OR "transcranial ultrasound" OR "focused ultrasound" OR "low-intensity focused ultrasound" OR "low intensity focused ultrasound" OR tFUS OR LIFU) | No language, publication-type, or date filters applied. | 392 |

**Search yield:** 454 database records were identified (Scopus, n = 392; Embase, n = 52; PubMed/MEDLINE, n = 10). After removal of 25 duplicates, 429 unique database records remained. Backward and forward citation searching identified three additional reports, yielding 432 records for screening.

**C. Complete data-extraction framework and variables collected**

| **Extraction domain** | **Variables collected** |
| --- | --- |
| **Study identification** | First author; publication year; country; journal/report type; study design; study setting. |
| **Participants** | Sample size; number randomized/enrolled/analyzed; age; sex distribution; disease duration; Parkinson’s disease phenotype where reported; Hoehn and Yahr stage; baseline disease severity; relevant comorbidities. |
| **Medication status** | Dopaminergic treatment; levodopa-equivalent daily dose when reported; stability of medication regimen; ON/OFF medication state during stimulation and outcome assessment. |
| **TPS intervention** | Device/system; stimulation target(s); laterality; targeting/neuronavigation method; anatomical distribution of stimulation; energy flux density; pulse repetition frequency; pulse duration when reported; pulses per session; session duration; number of sessions; treatment frequency; total treatment period; protocol individualization or dose adjustments. |
| **Comparator / sham** | Comparator type; sham procedure; within-participant or parallel comparison; treatment sequence; washout interval; blinding procedures where reported. |
| **Follow-up** | Assessment time points; immediate post-stimulation assessment; short-term and later follow-up; withdrawals and attrition. |
| **Motor outcomes** | UPDRS/MDS-UPDRS motor outcomes; tremor amplitude/frequency and clinical tremor measures; bradykinesia; rigidity; gait; balance; manual dexterity; reaction time; other validated motor outcomes. |
| **Non-motor and functional outcomes** | Non-motor symptom burden; cognition; depressive symptoms/mood; sleep; fatigue; activities of daily living; quality of life; freezing of gait; other patient-reported or functional measures. |
| **Neurophysiology** | EEG spectral measures and cortical oscillations; cortical excitability measures; motor-evoked potentials; other electrophysiological outcomes. |
| **Neuroimaging** | Task-based fMRI; resting-state fMRI; DTI; functional activation/connectivity; fractional anisotropy and other structural or white-matter measures. |
| **Safety and feasibility** | Adverse events; serious adverse events; discomfort/pain/pressure; treatment discontinuation; adherence; feasibility; acceptability; treatment-related structural MRI abnormalities where assessed. |
| **Effect estimates and statistics** | Baseline and follow-up values; direct active-versus-sham/comparator estimates when available; within-group changes when no valid between-condition estimate was available; effect sizes; confidence intervals when reported; p values; correction for multiple comparisons; duration/persistence of effects. |
| **Interpretive notes** | Principal study findings; important null findings; protocol-specific limitations; potential confounding; multicomponent interventions; relationship between clinical and biological outcomes. |
